# Crisis discharges and readmission risk in acute psychiatric male inpatients

**DOI:** 10.1186/1471-244X-8-44

**Published:** 2008-06-17

**Authors:** Dana JH Niehaus, Liezl Koen, Ushma Galal, Khalid Dhansay, Piet P Oosthuizen, Robin A Emsley, Esme Jordaan

**Affiliations:** 1 Department of Psychiatry, University of Stellenbosch, South Africa; 2 Biostatics Units of the Medical Research Council, Bellville, South Africa

## Abstract

**Background:**

Severe pressures on beds in psychiatric services have led to the implementation of an early ("crisis") discharge policy in the Western Cape, South Africa. The study examined the effect of this policy and length of hospital stay (LOS) on readmission rates in one psychiatric hospital in South Africa.

**Methods:**

Discharge summaries of adult male patients (*n *= 438) admitted to Stikland Psychiatric Hospital during 2004 were retrospectively examined. Each patient's clinical course was then analysed for the period between January 1^st^, 2004, and August 31^st^, 2006.

**Results:**

Although shorter LOS was associated with decreased readmission rates, the effect of crisis discharges was far more powerful. Patients discharged as usual had a far lower risk of readmission than those discharged due to bed pressures (i.e. crisis discharge).

**Conclusion:**

Increased risks associated with the early discharge policy necessitate the urgent review of the current management of bed shortages in this inpatient facility. The strengthening of community initiatives, particularly assertive outreach could be a way forward.

## Background

One of the global trends in psychiatric care in recent decades has been large scale deinstitutionalization, leading to a reduction in number of beds available and shorter length of hospital stay (LOS) for most patients [1,2]. Data from the US shows that psychiatric length of stay in all types of hospitals continued to decrease between 1988 and 1992 with it being most noticeable in psychiatric hospitals where length of stay declined from 75 to 56 days. In the same period there was a reduction of 12.5 million inpatient days in psychiatric hospitals in the US alone [3]. In the UK there is a similar trend with the number of in-patient psychiatric beds in England having fallen dramatically over the past four decades [4]. In addition to shortening of length of stay, Lay et al [1] also suggests an overall redistribution of treatment resources with decreased inpatient treatment for people with schizophrenia and an increase for affective disorders. This decrease in inpatient care for specific diagnostic categories is supported by at least some data from the US, that also suggests the decline in inpatient treatment has not led to an increase in outpatient visits for people with schizophrenia [5].

The shortening of length of stay seemed to be supported by early studies in the 1970's which showed no difference in readmission rates when comparing short vs. long term hospitalizations [6,7]. This was seen as evidence for the support of the deinstitutionalization process as it was suggested that longer hospitalization leads to difficulties for patients to re-enter the real world. The data examining the relationship between length of stay and rate of readmission have however not been unequivocal. For example a Cochrane meta-analysis could demonstrate no effect on readmission rates by planned short stay admissions, but the authors also stressed the need for more, large, well-designed trials, especially in the developing world [2].

Although reports on the consequences of the reduction in LOS have been ambiguous, what appears clear is that shorter LOS is only effective with proper discharge planning and outpatient care [8,9]. In the Western Cape Province, South Africa, an early "crisis" discharge policy has been adopted to deal with severe acute bed pressures [10] and thus impact on length of stay. Crisis discharge is defined as follows: "in the situation where an acutely mentally ill person in the community requires urgent admission to the hospital and no beds are available, clinical ward staff will identify an inpatient for early discharge. Since 2003 it has been standard operating procedure at Stikland Hospital to document on each patient's discharge summary whether they were a "crisis discharge" or not." Ideally the crisis discharged patient should meet the following criteria:

1. Most clinically stable patient in the ward

2. Not pose an immediate threat to him-/herself or others

3. Less ill than the patient that needs urgent admission

4. Most practical follow-up arrangements have been put into place prior to discharge

Despite the fact that changes in governmental policy and legislation are usually aimed at addressing shortcomings in the health care system, the impact of these changes are often not predictable. For example, Bauer et al. 2007 reported how changes in Israeli mental health policies ultimately led to an increase, rather than a decrease, of involuntary admissions [11].

Our aim was to determine the effects of the crisis discharge policy on readmission rates of acute psychotic male inpatients.

## Methods

### The crisis discharge policy

The 'crisis' discharge policy was adopted by the Associated Psychiatric Hospital management team in the Western Cape Province, South Africa, to deal with severe acute bed shortages in the state psychiatric sector [10].

### Study sample

This retrospective study (N05/03/047) was approved by the Committee for Human Research at the University of Stellenbosch, and patient confidentiality was protected by using only a study number linked to the particular patient. Data was captured by examining the discharge summaries of all male patients between the ages of 18 and 60 (extremes included) admitted and discharged from the acute wards at Stikland Psychiatric Hospital (SPH) in the year 2004. SPH is a state psychiatric facility with specialized wards for patients with acute psychosis. The genders are segregated and the facility has 80 such beds for male patients. As the bed shortage in the female wards is less pronounced, the emergency discharge policy is used much less and this study therefore focused on male patients only. It serves a mixed urban and rural community of 1.5 million people in the Western Cape Province of South Africa. Patients who were transferred to chronic psychotic or non-psychotic wards, to other hospitals, who self-discharged or died, were excluded from the analysis. Readmissions up until 31 August 2006 were included in the dataset. Data were captured using the patients' hospital folder number as reference and included variables for patient demographic characteristics, five axis DSM IV diagnosis, length of hospital stay (LOS) and discharge status (crisis discharge or discharge as usual).

### Statistical Analyses

Marital status was divided into 2 broad groups (either single or not single) and the diagnoses were divided into patients that had a co-morbid substance disorder diagnosis (HR = high risk) and those not having a co-morbid substance disorder diagnosis (LR = low risk). The time to readmission was calculated as the time between the first discharge and second admission, and was used as the outcome variable in the analysis. Crisis discharge and LOS were the main predictors.

**Figure 1:**
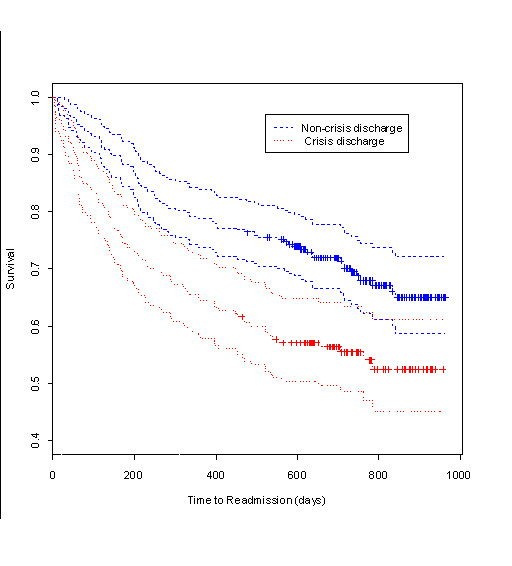
**Kaplan-Meier survival curves for crisis status**. The Kaplan-Meier curves showing the influence of crisis discharge on time to readmission. Crosses indicate where censoring took place. Plot includes 95% confidence intervals for the two Kaplan-Meier curves.

Summary statistics were initially produced for the data (Table 1) and Kaplan-Meier survival curves were generated. These are presented in Figure 1 and assume that the data is right-censored. The time to readmission variable was the time-to-event variable, while the 'event' was second admission. Patients not readmitted by 31 August 2006 formed the censored cases. These are represented by the crosses on the curves. Since the effect of crisis discharge on readmission was of interest, separate curves were produced for the crisis discharge and non-crisis discharge cases so that they could be compared.

**Table 1:** Summary statistics for index and readmission data

**Time to Readmission (days)**	**Median (n = 438)**	**Chi-Sq p-value**		
Crisis discharge group	628.0	0.007		
Non-crisis discharge group	687.5			
*LOS <= 39 days	693.5	<0.001		
LOS > 39 days	603.5			

**Re-admission data**				

	**Re-admission Yes (n = 163)**	**Re-admission No (n = 275)**	**Total (n = 438)**	**Chi-Sq p-value**

Crisis discharge group	81	99	180	0.004
Non-crisis discharge group	79	175	254	
Marital status: Single	139	217	356	-
Marital status: Not single	24	58	82	-
**Substance use disorder	20	28	48	-
High risk group				
Substance use disorder	19	38	57	-
Low risk group				
Income: FGP	1	1	2	-
Income: FMC	127	187	314	-
Income: FMS	18	22	40	-
Income: H1	16	60	76	-
Income: H2	1	1	2	-
Income: PMN	0	4	4	-
LOS <= 39 days	84	166	250	0.088
LOS > 39 days	79	109	188	

*LOS length of stay

** Substance use disorder high risk group fulfilled criteria for a substance use disorder and low risk group not.

***FGP defined as a person receiving a government pension, FMC = receiving a disability grant and certified to hospital, FMS = receiving a disability grant, H1 = income R 0.00 – R 3000.00 for individuals and R 0.00 – R 4100.00 for families, H2 = R 3001.00 – R 6000.00 for individuals and R 4101.00 – R 8330.00 for families, PMN = income above H2 levels.

To statistically test whether or not there is a difference in time to readmission between the crisis and non-crisis discharges, a Cox proportional hazards regression was carried out. The Cox regression model is a non-parametric model, which assumes that the hazard rate is a proportion. The model adjusted for LOS, marital status and income. Since there were patients that were not readmitted by the end of the focus period (31 August 2006), these observations were treated as right-censored data. Hazard ratios for were calculated from the results of the regression and are presented along with the corresponding confidence intervals in Table 2.

**Table 2:** Results of Cox Proportional Hazards Regression

	**Estimated Hazard Ratio**	**P-value**	**95% Confidence Interval**
Crisis Discharge	1.646	0.002	(1.200, 2.260)
Marital Status	1.416	0.130	(0.906, 2.210)
Income: FMC	1.119	0.920	(0.140, 8.970)
Income: FMS	1.189	0.870	(0.144, 9.840)
Income: H1	0.505	0.530	(0.060, 4.250)
Income: H2	1.272	0.870	(0.071, 22.940)
Income: PMN	NA	NA	NA
LOS	1.004	0.022	(1.001, 1.010)
Wald test = 26.6 on 7 df, p = 0.000391			

*LOS length of stay

** Substance use disorder high risk group fulfilled criteria for a substance use disorder and low risk group not.

***FGP defined as a person receiving government pension, FMC = receiving a disability grant and certified to hospital, FMS = receiving a disability grant, H1 = income R 0.00 – R 3000.00 for individuals and R 0.00 – R 4100.00 for families, H2 = R 3001.00 – R6000.00 for individuals and R 4101.00 – R 8330.00 for families, PMN = income above H2 levels.

The statistical analyses were done using the package R: A Language for Data Analysis and Graphics (freely available [12]), SAS Enterprise Guide and the Software Package for Social Sciences [13].

## Results

### Demographics

The participants' mean age on admission was 32.9 (s.d. = 10.4) years. Most patients (*n *= 356 or 77%) belonged to the group made up of single, divorced or widowed people. The majority of the patients were primarily Afrikaans speaking (*n *= 359 or 82.3%). Of the DSM IV axis I diagnoses, *n *= 307 (70.0%) were accounted for by just three diagnoses; Schizophrenia (*n *= 207 or 47.2%), Bipolar Disorder (*n *= 67 or 15.3%) and Schizo-affective disorder (*n *= 33 or 7.5%). Of these, 119 (38.9%) exhibited a co-morbid substance-related disorder. The majority of patients (*n *= 339 or 77.2%) were involuntary admissions and 15% (*n *= 67) were assisted users (user not refusing admission but currently regarded as not competent to judge treatment needs).

### Readmissions and Crisis Discharge

Of the 438 admissions included in the analysis, 180 patients (41.0%) were crisis discharges on their first discharge, whilst 254 (58.0%) were discharged as usual (missing data for four patients). The mean LOS for all admissions was 43.9 (s.d. = 39.4) days. For the crisis discharges, the mean LOS was 40.6 (s.d. = 32.7) days, while for the non-crisis discharges it was 46.4 (s.d. = 43.7) days. The median time to readmission was longer (688 days) for the non-crisis discharge group than for the crisis discharge group (628 days).

During the entire study period, 163 (37.2%) of the 438 index admissions were readmitted to hospital. This means that more than half the observations, 275 (62.8%) were censored. Of the readmissions, 81 (50.6%) were crisis discharge patients. 45% of the crisis discharge group were readmitted, while the same was true of 31% of the non-crisis discharge group.

### Time to readmission

If we refer to Fig. 1, we see that there is (visually) a difference between the survival functions of the crisis and non-crisis discharge groups. The non-crisis discharge group has a higher survival time, where survival time is the time to readmission. The graph includes 95% confidence intervals which generally support the result that the two groups are different.

The Cox regression results in Table 2 show that crisis discharge had a significant influence on the time to readmission (Hazard ratio = 1.646, *P *= 0.002, *CI *1.200, 2.260). A hazard ratio of 1.646 implies that the hazard for the crisis group is 1.6 times that of the non-crisis discharge group, so the crisis discharges will be readmitted sooner than those that received a complete treatment. The table also shows that marital status and income had no influence on time away from the hospital until readmission. As expected, LOS had a marginally significant effect on time away from the hospital (Hazard ratio = 1.004, *P *= 0.022 *CI *1.001, 1.010). LOS and crisis discharge did not interact significantly with each other, nor did any of the other factors interact significantly.

## Discussion

### Impact of crisis discharge

Our study showed that so-called "crisis discharge" was associated with a significantly increased risk of readmission and shorter time until readmission and this was independent of the impact that LOS had on readmission rates. The link between increased risk of readmission and crisis discharges was not unexpected, considering that such discharges are, by definition, sudden and unplanned. In South Africa and internationally, maintaining patients in the community, prevention of relapse and reduction of risk of readmission are often the responsibilities of the community psychiatric services [14,15]. Patients who are discharged as usual are more likely to be better engaged and more likely to follow up with outpatient care [16]. Furthermore, a pre-discharge program makes it easier to address issues such as co-morbid substance use or abuse [17], which was highly prevalent in our sample (27.1%) although it did not increase readmission risk.

### Length of hospital stay

In this study, we found that decreased LOS led to longer time until readmission. However, the results in Tables 1 &2 are at best borderline and therefore inconclusive. Figueroa *et al*[16] found a direct relationship between decreasing LOS and readmission rates when examining private psychiatric inpatients. Similarly, Appleby *et al*[18] found higher readmission rates in schizophrenic patients with a LOS of less than 30 days compared to those with a length of stay of more than 30 days in a public psychiatric hospital. Heeren *et al*[19] as well as Wickizer *et al*[20] found a positive correlation between shorter LOS and readmission rates in a geriatric and child and adolescent unit respectively. It has therefore been postulated that too short a LOS does not allow for a resolution of the patient's clinical condition nor allow adequate preparation for the patient's discharge, thereby contributing to a revolving door effect [9,21].

However, a number of other studies [2,8,22] did not find any adverse outcomes for short hospital stays. It is not surprising that studies examining length of stay and readmission rates have not always reported consistent findings. There is a large variability in reported lengths of stay in different studies, with private institutions reporting a mean LOS of 7.1 and 6.7 days [14,16] and psychiatric hospitals a mean LOS of 50.0 and 63.2 days [23,24]. The latter compares more closely with ours of 44 days. The results from these studies are also influenced by factors such as different hospital types (general versus psychiatric), sources of funding (state versus private), differing study populations (adult, older and young adults) and the large variability in psychiatric diagnoses, natural course and response to treatment. Importantly, in many of these studies, short hospital stays were often pre-planned [22,25,26]. Also, in the case of private psychiatric hospitals the length of stay is usually predetermined at the time of admission which might stress the importance of discharge planning irrespective of the LOS. This differs from the crisis discharge, which is, by definition, sudden and therefore unplanned.

### Implications

Although our one-year readmission rate of 15.5% can be favourably compared to Lyons *et al*'s [14] six-month readmission rate of 17.6% and Segal *et al*'s [9] one-year readmission rate of 29%, we present evidence that the crisis discharge policy may exacerbate a revolving door effect in Stikland psychiatric hospital. Such frequently-returning patients may contribute significantly to costs and bed-occupancy, thereby counteracting the intended cost reductions that were the motivation for, inter alia, shorter LOS. In addition, the clinical management of frequently admitted patients may be adversely affected through demotivation of staff and therapeutic nihilism [27] if readmitted patients are viewed as 'regulars' who have familiar, unchanging repetitive issues and patterns of admissions within an already struggling and short-staffed mental health system.

### Strengths and limitations

A major strength of this study was that all admissions were from one hospital and data/participants were evaluated in a standardized fashion. This yielded a sizeable study sample that was followed up over a long period, comparing very favourably to sample sizes and study period of other studies [e.g [14]]. The use of one treatment facility lessened the impact of physician variability on the use of the crisis discharge policy and the retrospective nature of the trial reduced any potential bias regarding LOS. The impact of patient variables were also lessened due to the large variability of patients within our drainage area, which included both involuntary and voluntary, state and medical aid patients from different ethnic and socio-economic backgrounds.

Staggered admissions may have had an impact on readmission rates and a longitudinal follow-up design may uncover valuable data obscured by our current analysis. Admission data prior to the implementation of the crisis discharge policy, which could have strengthened this study substantially, was not available. This is especially significant if the total number of admissions over the last few years had increased without a concomitant increase in the number of inpatient beds, as this would have a direct bearing on the number of crisis discharges for the year. Finally, readmissions would have been missed if patients were admitted to a private or other psychiatric hospital, but this would have introduced a positive bias to our results.

## Conclusion

This is the first study to address the possible impacts of the Western Cape Province's APH crisis discharge policy on patient rehabilitation and readmission. Further research is clearly needed on the implications of these findings as well as ways of reviewing crisis discharge policies and its adverse outcome on readmissions. LOS and the crisis discharge policy seem to exacerbate the revolving door effect in this psychiatric hospital. Readmission is often used as quality indicator for inpatient psychiatric services, and could be seen as a failure of the earlier hospital admission especially when it occurs within a relatively short-time after a previous discharge. Since the main factor influencing the crisis discharge policy is inpatient bed availability, provision for increasing the number of available acute psychiatric beds needs to be seriously considered in an effort to reduce the incidence of crisis discharges. Additionally, or alternatively, strengthening community based services, particularly outreach initiatives, would be important and we believe this to be the way forward.

## Competing interests

The authors declare that they have no competing interests.

## Authors' contributions

KD, LK and DJHN conceived of and designed the study. KD acquired the data. UG and EJ performed the statistical analysis. DJHN, LK, PPO and RAE drafted the manuscript. All authors read and approved the final manuscript.

## Pre-publication history

The pre-publication history for this paper can be accessed here:


